# Progressive respiratory distress in a 42-year-old HIV-positive woman with systemic lupus erythematosus

**DOI:** 10.1186/s40001-017-0261-1

**Published:** 2017-06-17

**Authors:** Katongo Mutengo, Patrice Mukomena, Nason Lambwe, Owen Ngalamika

**Affiliations:** 1 Department of Medicine, University Teaching Hospital, University of Zambia School of Medicine, Lusaka, Zambia; 2 Dermatology and Venereology Section, Department of Medicine, University Teaching Hospital, University of Zambia School of Medicine, Lusaka, Zambia

**Keywords:** Systemic lupus erythematosus, HIV, Pulmonary embolism, Respiratory distress

## Abstract

**Background:**

Identifying and treating the cause of pulmonary symptoms in HIV patients with underlying systemic lupus erythematosus (SLE) can be very challenging. Delays in diagnosing active SLE in HIV patients can lead to significant morbidity and even mortality. We report the case of an HIV-positive woman with SLE who presented with severe respiratory distress.

**Case presentation:**

A 42-year-old HIV-positive woman presented with a 7-month history of anorexia, progressive dyspnoea, and a productive cough. She had been put on treatment for pulmonary tuberculosis and pneumocystis jiroveci pneumonia for several months by the referring hospital without any significant improvement in her symptoms. Her initial laboratory investigations showed highly elevated d-dimer test results but confirmatory investigations for pulmonary embolism proved otherwise. An autoimmune screen revealed highly positive antinuclear antibody and anti-double-stranded DNA tests, and she responded very well to SLE treatment.

**Conclusions:**

Our case represents a situation where two diseases with antagonizing pathways of disease pathogenesis occur concurrently in the same patient. SLE is usually not among the differential diagnoses in HIV patients with respiratory distress. Management of patients with both SLE and HIV is also very challenging because improvement in one condition can lead to worsening of the other. Despite opportunistic infections being the likely cause of pulmonary symptoms in HIV patients, clinicians are encouraged to have a high index of suspicion for autoimmune interstitial lung disease in these patients.

## Background

Systemic lupus erythematosus (SLE) is an autoimmune disorder which can affect any organ system of the body. Its diagnosis is often challenging as it is a multifaceted disorder and the presentation varies from patient to patient [1]. SLE patients commonly present with constitutional symptoms coupled with skin, musculoskeletal, and/or renal involvement [2]. Coexistence of SLE and human immunodeficiency virus (HIV) presents a diagnostic challenge as both conditions may have a similar clinical course [3]. In HIV-endemic areas, the paradigm is often shifted toward the screening and diagnosis of common opportunistic infections. Therefore, screening for autoimmune disorders in HIV patients is remotely done.

There are many plausible diagnoses in an HIV-positive woman presenting with pulmonary symptoms in sub-Saharan Africa. SLE or other autoimmune lung diseases are usually not on the list of differential diagnoses. In such cases, pulmonary tuberculosis (PTB) and *Pneumocystis jiroveci* pneumonia (PCP) are among the most prevalent underlying conditions. Furthermore, in an HIV-infected patient with some clinical and laboratory findings suggestive of pulmonary embolism (PE), it is even more difficult to entertain the possibility of SLE. We report the case of an HIV-positive woman with SLE who presented with symptoms and signs of pulmonary disease in the absence of typical clinical features of SLE.

## Case presentation

A 42-year-old HIV-positive Zambian woman of African descent presented to the emergency department with a 7-month history of anorexia, progressive dyspnoea, and a productive cough with mucoid sputum. The patient had a history of receiving at least 6 months of anti-tuberculosis treatment (ATT) from her referring hospital based on symptoms and chest radiographic findings, without any significant clinical improvement. In addition, she received high-dose co-trimoxazole for suspected *Pneumocystis jiroveci* pneumonia (PCP) which was also without notable effect. At the time of presentation, she had been on combined anti-retroviral treatment (cART) for about 6 years. She denied any history of rash, joint pains, headaches, or photophobia.

On examination, she appeared ill, was fully conscious, apyrexic, tachypneic (32 breaths per minute), and tachycardic (120 beats per minute) with a peripheral oxygen saturation of 84% on ambient air and a blood pressure of 110/70 mmHg. On auscultation, coarse extensive crackles were heard in both lung fields and the heart sounds were regular with a loud P2 noted in the tricuspid area. The examination was negative for any skin rash, alopecia, joint swelling/deformity, hepatosplenomegaly, ascites, or peripheral edema.

**Fig. 1:**
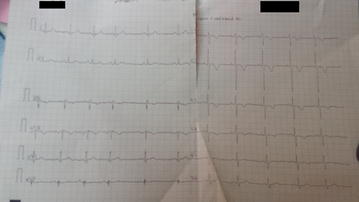
Initial ECG. Sinus tachycardia with *left axis* deviation, *right ventricular* strain pattern with T wave inversion in V1–V4 as well as lead III, and prominent R wave in right-sided leads

An initial electrocardiogram showed a sinus tachycardia with left axis deviation, right ventricular strain pattern with T wave inversion in V1–V4 as well as lead III, and prominent R wave in right-sided leads which were in keeping with right ventricular hypertrophy (Fig. 1). Her chest X-ray showed an enlarged cardiac shadow with air bronchograms and diffuse reticular shadowing. An urgent transthoracic echocardiogram revealed a mild pericardial effusion, right atrial and right ventricular enlargement with tricuspid regurgitation, and pulmonary hypertension. Initial blood gases showed a picture of hypoxemia with low arterial carbon dioxide consistent with type 1 respiratory failure pattern (PH 7.46, PCO_2_-27 mmHg; expected compensation of 34–38 mmHg by Winters’ equation, PO_2_-42 mmHg, HCO_3_-18.7 mEq/L). D-dimers were markedly elevated at 2600 ng/mL. Despite the highly elevated d-dimer levels, the follow-up chest CT was negative for pulmonary embolism, and the findings were reported as normal. A Doppler ultrasound of both lower limbs excluded venous thromboembolism. Her abdominal ultrasound scan was also normal. Sputum for Gene-Xpert, microscopy, culture, and sensitivity were obtained to exclude other infectious etiology. The working diagnosis at this point was subacute pulmonary embolism (PE) to exclude chronic infectious pneumonia. The patient received high-flow oxygen at 8 L/min via face mask, low-molecular weight heparin (enoxaparin 80 mg twice daily), warfarin (5 mg once daily), and furosemide 40 mg once daily in view of extensive fine crackles in both lung fields. Results of the investigations performed are shown in the table below (Table 1).

**Table 1:** Results of the initially ordered laboratory investigations

Laboratory parameter	Result	Reference range
White cell count	10.5 × 10^9^/L	4.0–11.0 × 10^9^/L
Hemoglobin	12.4 g/dL	11.5–16.5 g/dL
Platelets	330 × 10^9^/L	150–400 × 10^9^/L
Lactate dehydrogenase	*494* *IU/L*	240–480 IU/L
Liver function tests	Normal	
Renal function tests	Normal	
Thyroid function tests	Normal	
Sputum microbiology	Negative	
Urine microbiology	Negative	
INR	*1.3*	2.0–4.0
Serum ACE levels	24.03 U/L	9.0–55.0 U/L
Serum ionized calcium	9.10 mg/dL	8.1–10.4 mg/dL
CD4 count	639 cells/µL	500–1200 cells/µL
ANA+	*1:100* and *1:200*	
Anti-dsDNA	*37.6* *U/mL*	<20 U/mL
CRP	*89* *mg/dL*	<10 mg/dL
D-dimers	*2600* *ng/mL*	<500 ng/mL
Anti B2 glycoprotein 1	Negative	
Lupus anticoagulant	Negative	
Anti-cardiolipin	Negative	

Italics represent abnormal values

The patient improved during the course of admission. She was no longer oxygen dependent, her SaPO_2_ was 94% on ambient air, the lung fields were clear on auscultation, and there was a reduction in her respiratory rate to 24/min. Her sputum investigations for tuberculosis and bacterial infection were negative. She was discharged on day 9 based on the pulmonary embolism severity index (PESI) low-risk score on warfarin 5 mg once daily, pending laboratory results for repeat d-dimers. However, she was readmitted 5 days later due to the recurrence of severe respiratory distress. On re-admission, she was very restless, breathless at rest, and apyrexic. She was neither pale nor cyanosed. Her peripheral saturation was 98% on 4 L/min of oxygen via the nasal cannula. She had no features of fluid overload. The lung fields were clear on auscultation but of note was a pericardial rub and loud P2. Meanwhile, repeat transthoracic echocardiogram showed a mild pericardial effusion and pulmonary hypertension. An electrocardiogram was repeated and had features consistent with the first one done on the initial admission.

There was normalization of her d-dimers (396 ng/mL). However, the test results for autoimmune diseases showed a strongly positive ANA and anti-dsDNA. Anti-histone and anti-phospholipid antibodies (anti-β2 glycoprotein 1, lupus anticoagulant, and anti-cardiolipin antibodies) were negative. Based on the results, a diagnosis of active SLE with a possibility of chronic lupus interstitial lung disease was made. The patient was admitted in a high dependency ward for further treatment and support. She received intravenous methylprednisolone 500 mg stat, then 250 mg once daily for 4 days followed by oral prednisolone 50 mg once daily. She also continued her warfarin therapy. She was discharged 11 days later in an asymptomatic state on prednisolone, hydroxychloroquine, omeprazole, and warfarin. On her scheduled review in the rheumatology clinic a fortnight later, she remained asymptomatic and a slow tapering down of prednisolone was begun. She continued to live a normal life. In view of her HIV status and steroid use, routine immunological assessment was scheduled for every 3 months as well as monthly medical reviews for warfarin therapy and INR monitoring.

## Discussion

Coexistence of HIV and SLE often poses a diagnostic and treatment dilemma. Few cases have been reported of both HIV infection and SLE in the same individual [1, 3]. Evidence suggests that clinical features of HIV can mimic those of SLE and vice versa [4]. In an HIV-infected individual, the diagnosis of active SLE presenting with purely pulmonary features becomes even more arduous. Our patient presented with features mimicking an acute pulmonary condition. At the primary health care before she presented to our tertiary institution, she was treated presumptively as a case of PCP in view of severe respiratory distress in HIV. However, she did not respond to several months’ treatment with high-dose co-trimoxazole. Furthermore, due to her HIV status, a diagnosis of pulmonary tuberculosis was also entertained, for which she received ATT without significant clinical improvement. The poor response to anti-tuberculosis and PCP treatments, negative results for a possible infectious cause, a high CD4 count, recurrence of symptoms despite initial improvement on PE treatment plus cardiac findings (pericardial rub and loud P2) led us to entertain the possibility of an underlying autoimmune condition.

It has been reported that the immunosuppression associated with HIV infection may lead to a decline in autoantibody production and therefore may improve the clinical symptoms of SLE [5]. On the other hand, SLE patients may produce antibodies that may be able to control HIV viral replication [6, 7]. This interplay shows that SLE and HIV may have a protective effect on each other. Our patient was doing well immunologically as far as HIV infection is concerned. This may have led to a loss of immune homeostasis and flare-up in SLE.

The dilemma in managing our patient was commencing her on high-dose immunosuppressive therapy, which was the best for managing SLE symptoms but could potentially increase HIV replication and hence promote rapid progression of the HIV clinical course [8]. However, the patient was closely followed up, with routine CD4 counts done at 3-month intervals, and adherence to ART was repeatedly emphasized.

We had a high index of suspicion for PE in this patient before alternative diagnosis was sought. In favor of this was a moderate pretest PE Wells score of 4.5 (HR >100 bpm and lack of alternative diagnosis to explain illness), hypoxia and hypocapnia, high d-dimers, and ECG findings of right ventricular strain pattern with T wave inversion in V1–V4 as well as lead III (described in 34% of PE). This prompted us to also screen for anti-phospholipid antibodies which came out negative. Anti-phospholipid antibodies have been known to be associated with PE through promotion of thrombosis [9]. The ECG, echocardiogram, chest CT, and lower limb Doppler ultrasound scan further reduced the likelihood of PE. However, a more sensitive investigation such as CT pulmonary angiography, if available, could have provided much more accurate diagnostic information on PE. In addition, a positive d-dimer test is not a very reliable marker because it is not specific for PE. Many factors, including SLE, are known to be associated with a positive d-dimer test [10]. Nevertheless, optimal treatment for PE was administered in view of some supporting physical signs and investigations, and to prevent a fatal outcome associated with untreated PE. Even after improvement in d-dimers to normal levels on anticoagulation therapy, we did not disregard PE as a potential coexisting condition but continued the patient on warfarin therapy guided by a target INR of 2–3.

SLE presents with various pulmonary manifestations which could partially explain the patient’s symptoms. These include chronic interstitial pneumonitis, acute lupus pneumonitis, pulmonary vascular disease, airway disease, and infectious complications [11]. In some instances, SLE may lead to false-positive HIV test results by western blot [12]. For this reason, the HIV-positive result in this patient was confirmed by DNA PCR.

We were unable to perform further diagnostic investigations for PE such as ventilation/perfusion (V/Q) scan and CT pulmonary angiography due to unavailability of such facilities. Nevertheless, our patient responded very well to SLE treatment which further supported our final diagnosis. The initial response to anticoagulant therapy and high d-dimers makes it very difficult to completely rule out the possibility of PE in our patient despite the negative anti-phospholipid antibody test and chest CT scan. Therefore, it is possible that PE could have caused the sudden exacerbation of SLE interstitial lung disease in our patient.

## Conclusions

Pulmonary manifestations are many and very common in HIV patients. Clinicians are encouraged to have a high index of suspicion for autoimmune interstitial lung disease in HIV patients presenting with pulmonary symptoms that cannot be explained by infectious causes or are recalcitrant to treatment for opportunistic infections.

## Abbreviations

ANA: antinuclear antibody
ART: anti-retroviral therapy
ATT: anti-tuberculosis treatment
cART: combined anti-retroviral therapy
CT: computed tomography
DNA: deoxyribonucleic acid
DNA PCR: deoxyribonucleic acid polymerase chain reaction
ECG: electrocardiogram
HIV: human immunodeficiency virus
PCP: *Pneumocystis jiroveci* pneumonia
PE: pulmonary embolism
SLE: systemic lupus erythematosus
